# Accuracy of chimeric proteins in the serological diagnosis of chronic chagas disease – a Phase II study

**DOI:** 10.1371/journal.pntd.0005433

**Published:** 2017-03-08

**Authors:** Fred Luciano Neves Santos, Paola Alejandra Fiorani Celedon, Nilson Ivo Tonin Zanchin, Wayner Vieira de Souza, Edimilson Domingos da Silva, Leonardo Foti, Marco Aurélio Krieger, Yara de Miranda Gomes

**Affiliations:** 1 Aggeu Magalhães Research Center, Oswaldo Cruz Foundation, Recife, Pernambuco, Brazil; 2 Chagas Disease Integrated Program, Oswaldo Cruz Foundation, Rio de Janeiro, Rio de Janeiro, Brazil; 3 Molecular Biology Institute of Paraná, Curitiba, Paraná, Brazil; 4 Carlos Chagas Institute, Oswaldo Cruz Foundation, Curitiba, Paraná, Brazil; 5 Biomanguinhos, Oswaldo Cruz Foundation, Rio de Janeiro, Rio de Janeiro, Brazil; Academic Medical Centre, NETHERLANDS

## Abstract

**Background:**

The performance of current serologic tests for diagnosing chronic Chagas disease (CD) is highly variable. The search for new diagnostic markers has been a constant challenge for improving accuracy and reducing the number of inconclusive results.

**Methodology/Principal findings:**

Here, four chimeric proteins (IBMP-8.1 to -8.4) comprising immunodominant regions of different *Trypanosoma cruzi* antigens were tested by enzyme-linked immunosorbent assay. The proteins were used to detect specific anti-*T*. *cruzi* antibodies in the sera of 857 chagasic and 689 non-chagasic individuals to evaluate their accuracy for chronic CD diagnosis. The antigens were recombinantly expressed in *Escherichia coli* and purified by chromatographic methods. The sensitivity and specificity values ranged from 94.3% to 99.3% and 99.4% to 100%, respectively. The diagnostic odds ratio (DOR) values were 6,462 for IBMP-8.1, 3,807 for IBMP-8.2, 32,095 for IBMP-8.3, and 283,714 for IBMP-8.4. These chimeric antigens presented DORs that were higher than the commercial test Pathozyme Chagas. The antigens IBMP-8.3 and -8.4 also showed DORs higher than the Gold ELISA Chagas test. Mixtures with equimolar concentrations were tested in order to improve the diagnosis accuracy of negative samples with high signal and positive samples with low signal. However, no gain in accuracy was observed relative to the individual antigens. A total of 1,079 additional sera were used to test cross-reactivity to unrelated diseases. The cross-reactivity rates ranged from 0.37% to 0.74% even for *Leishmania* spp., a pathogen showing relatively high genome sequence identity to *T*. *cruzi*. Imprecision analyses showed that IBMP chimeras are very stable and the results are highly reproducible.

**Conclusions/Significance:**

Our findings indicate that the IBMP-8.4 antigen can be safely used in serological tests for *T*. *cruzi* screening in blood banks and for chronic CD laboratory diagnosis.

## Author summary

Chagas disease infection is a debilitating and neglected disease that occurs in 21 Latin America countries. The laboratory diagnosis is based on finding specific antibodies against *Trypanosoma cruzi* pathogen. Several methods are available currently, but their performance depends on matrix used as antigen. In general, two methodologies are required to perform a correct diagnosis. We tested four chimeric proteins comprised by various antigens of *T*. *cruzi* by a method designated enzyme-linked immunosorbent assay. The proteins were high sensitivities and specifics, presenting accuracy higher than the commercial test Pathozyme Chagas. Additionally, the proteins IBMP-8.3 and -8.4 were more accurate than the Gold ELISA Chagas test. The proteins presented a very low cross-reactivity to unrelated diseases, including *Leishmania* spp. Reproducibility analyses showed that IBMP proteins are stable and the results are highly reproducible. Our findings indicate that the IBMP-8.4 protein can be safely used in serological tests for *T*. *cruzi* screening in blood banks and chronic CD laboratory.

## Introduction

Human Chagas disease (CD) is a vector-borne neglected tropical disease caused by a hemoflagellated protozoan parasite called *Trypanosoma cruzi*, which is responsible for significant disease burden in 22 endemic countries in Latin America [1]. Infection with *T*. *cruzi* results in 14,000 deaths every year [1] and morbidity in 5.7 to 9.4 million people in the continental Western Hemisphere [2,3]. Since the late ‘90s, population shifts and migration have progressively favored the spreading of infected people worldwide transforming the disease into a global health concern, especially in North America, Europe, and Oceania countries [4–6].

Diagnosis of CD infection is not simple. During the acute phase, which is short and mostly asymptomatic, the parasites can easily be found in the blood of infected individuals. Therefore direct microscopic observation has been the chosen method for diagnosis. In contrast, indirect immunological methods present low sensitivity in recently infected individuals because the humoral response is delayed [7]. The chronic phase begins 8–10 weeks after the acute phase and may last for several years or even for the lifetime of the host. Owing to low parasitemia with high levels of specific anti-*T*. *cruzi* antibodies, diagnosis at this stage requires the utilization of antibody-antigen methods, including indirect hemagglutination (IHA), indirect immunofluorescence (IIF), rapid diagnostic tests (RTDs), and enzyme-linked immunosorbent assay (ELISA). The latter is the most commonly used due to its simplicity and the ease of automation. However, its performance depends on the antigen preparations used to detect the anti-*T*. *cruzi* antibodies [8,9]. Whole-cell homogenates or fractionated lysates of *T*. *cruzi* at the epimastigote stage have been used as complex mixtures of antigens for detecting *T*. *cruzi* infection. Although these combinations have been shown to render appropriate sensitivity to detect even low antibody levels [10], difficulties in standardizing the method, cross-reactivity, and specificity problems have been described and hinder their utilization [9,11–13].

Advances in DNA recombinant technology have allowed using recombinant proteins as sensitizing agents in ELISA assays since large quantities can be obtained in a purified form [14]. About 25% of *T*. *cruzi* expressed sequences contain tandem repeat motifs composed of 5–68 amino acids. This results in enhanced recognition by antibodies as opposed to non-repetitive proteins [11,15,16] and increases their performance in immunoassays compared to cell extracts [14,17]. The sera of infected individuals frequently contain high titers of antibodies against these repetitive sequences [18–20]. However, it has been observed that tests using these proteins can produce a certain number of false negative results. The high genetic variability of *T*. *cruzi* could be partly responsible for false negatives due to low antigen sequence representation in the tandem repeat motifs. To avoid this limitation, several studies have described the combined utilization of two or more recombinant proteins in a single test to increase sensitivity without loss of specificity [21–25]. This strategy could affect the performance of the test due to unbalanced binding of these epitopes to the solid surface, competition for binding, and spatial distribution of epitopes in the solid phase. However, ELISA tests including a mix of fusion proteins have been provided a good performance (Abbott PRIM [26]). More recently, an array of distinct antigens printed in each well of 96-well plates showed potential to diagnosis chronic CD [27].

A new strategy was developed that combines the characteristics of both standard procedures. Synthetic chimeras composed of repetitive fragments of amino acid of several antigenic proteins of the parasite have been proposed to improve the assay’s accuracy to diagnose chagasic infection [14,28,29]. Recently, our team evaluated four chimeric proteins for detection of antibodies against *T*. *cruzi* in human serum [9]. In that study, we established the optimal experimental conditions for both ELISA and liquid microarray using the chimeras IBMP-8.1, IBMP-8.2, IBMP-8.3, and IBMP-8.4. Under the optimized conditions, all antigens discriminated appropriately between CD-negative and chronic CD-positive samples. The high accuracy observed for all proteins motivated us to perform a Phase II study using serum samples from different geographical endemic regions of Brazil and other endemic countries. The findings of these analyses are presented in this study. The use of statistical tools allowed a robust assessment of the performance of each molecule.

## Materials and methods

### Ethics statements

Approval was granted by the Institutional Review Board (IRB) for Human Research at the Aggeu Magalhães Research Center, Oswaldo Cruz Foundation (FIOCRUZ), Recife, Pernambuco (PE), Brazil (CAEE: 15812213.8.0000.5190), with strict adherence to the principles laid out in the Declaration of Helsinki. Samples were obtained from the sera banks of the Reference Laboratory for Chagas Disease (FIOCRUZ-PE), Hemope Foundation-PE, LACEN-PE, Laboratory for Research on Chagas Disease from Federal University of Goiás (GO, Brazil) and Molecular Biology Institute of Paraná (PR, Brazil). To protect each patient’s identity, the IRB determined that all samples were to be anonymized so that the researchers would not have access to the patients’ private data, thereby avoiding the need for verbal or written consent.

### Recombinant chimeric protein production

Immunodominant sequences were selected, synthetic genes were constructed, and recombinant chimeric proteins were expressed according to the methods prescribed by Santos et al. [9] (further information see S1 Table). Briefly, synthetic genes encoding *T*. *cruzi* chimeric antigens were obtained from a commercial supplier (GenScript, Piscataway, NJ, USA) and subcloned into the pET28a vector. Chimeric antigens were expressed as soluble proteins in *Escherichia coli* BL21-Star (DE3) grown in LB medium supplemented with 0.5 mM of isopropyl β-D-1-thiogalactopyranoside. Proteins were first purified by both affinity and ion exchange chromatography, then quantified using a fluorimetric assay (Qubit 2.0, Invitrogen Technologies, Carlsbad, CA, USA).

### Sample size and human serum collection

Sample size was computed with a 95% confidence interval, expected sensitivity and specificity of 99%, and absolute error of 2%. Based on these parameters, obtained with program R [30], the minimum sample to carry out this study was 380 sera from chagasic (Ch) and 380 sera from non-chagasic (NCh) individuals. A total of 2,625 previously collected anonymized human serum samples were used to evaluate the performance of IBMP chimeras for *T*. *cruzi* by ELISA. Sera were obtained from NCh (n = 630) and Ch (n = 825) individuals from a variety of Brazilian endemic and non-endemic areas, including Bahia (BA), Minas Gerais (MG), Goiás (GO), Pernambuco (PE), and Paraná (PR). Additionally, 91 sera from chagasic and non-chagasic patients from Brazil, the United States, Mexico, Nicaragua, Guatemala, Honduras, and Argentina, were obtained from the National Panel for Blood Screening Quality Control (Fiocruz/RJ, Brazil), SeraCare Life Sciences Inc. (Milford/MA, USA), and Boston Biomedical Inc. (Norwood/MA, USA). In addition to these chagasic and non-chagasic sera, 1,079 samples from patients with unrelated diseases, as previously defined by their serological or parasitological diagnoses, were incorporated into the present sera sample set to evaluate cross-reactivity. The unrelated diseases evaluated included dengue (n = 50), filariasis (n = 51), hepatitis B virus (n = 163), hepatitis C virus (n = 98), HIV (n = 140), HTLV (n = 109), *Leishmania* spp. (n = 153), leptospirosis (n = 92), measles (n = 21), rubella (n = 15), schistosomiasis (n = 42), and syphilis (n = 145). All serum samples were serologically retested for antibodies against *T*. *cruzi* using two ELISA assays: Imuno-ELISA Chagas (Wama Diagnóstica-SP, Brazil, batch 14D061) and ELISA Chagas III (BIOSChile, Ingeniería Genética S.A., Santiago, Chile, batch 1F130525). Samples that returned discordant results, or those that were judged as inconclusive were excluded. Each sample was given a unique identifier code to ensure a blinded analysis.

### In-house ELISA

Anti-*T*. *cruzi* serology was carried out by ELISA as previously described [9]. In all experiments, background values were subtracted from the measurement tests.

### Imprecision assessment

Imprecision was assessed by within-run, between-lot, and between-lab testing using the Ch and NCh samples obtained in Pernambuco-Brazil. Within-run imprecision (or repeatability) was estimated by evaluating each specimen (23 chagasic and 23 non-chagasic) twice sequentially within the same run. Between-lot and between-lab imprecision was estimated using 45 Ch and 46 NCh, and 38 Ch and 46 NCh sera, respectively. Between-lab tests were performed at the Reference Laboratory for Chagas Disease (FIOCRUZ-PE), designated Lab 1, and at the Laboratory of Diagnostic Technology (FIOCRUZ-RJ), designated as Lab 2.

### Commercial ELISA test comparison

A total of 857 Ch and 689 NCh samples were selected to perform comparisons with respect to performance and agreement among the IBMP chimeric antigen assays and the commercial *T*. *cruzi* ELISA tests. Commercial test selection was based on Technical Note N°03/06 issued by the Brazilian Health Ministry [31], which evaluates the performance of commercial kits to diagnose Chagas in Brazil. Accordingly, the following two commercial Chagas disease-specific enzyme immunoassays were selected: Pathozyme Chagas (batch 7042779; Omega Diagnostics, Scotland, United Kingdom), which is based on recombinant antigens; and Gold ELISA Chagas (batch CHA132A; Rem, São Paulo, Brazil), which uses both recombinant antigens and lysates from the epimastigotes of *T*. *cruzi* strains found in Brazil.

### Statistical analysis

Data were encoded, analyzed, and presented using scatter plot graphing software (GraphPad Prism version 6, San Diego-CA, USA). Descriptive statistics are presented as geometric mean ± standard deviation (SD). Data set normality was tested using the Shapiro-Wilk test, followed by the Student’s t-test, and when homogeneity assumption was not confirmed, the Wilcoxon signed-ranks test was applied. All analyses were two-tailed, and a p-value of less than 5% was considered significant (p-value < 0.05). Cut-off point analysis was used to identify the optimal value of optical density (OD) to differentiate between negative and positive samples. The threshold value was established by measuring the longest distance from the diagonal line formed by the endpoints of the receiver operating characteristic curve (ROC) [sensitivity x (1-specificity)]. All results were expressed by plotting the values in an index format representing the ratio between a given sample’s OD and the cut-off OD pertaining to each microplate. This index is referred to as a reactivity index (RI) and all results < 1.00 were considered negative. If a sample’s RI value was 1.0 ± 10%, it was considered as indeterminate (or in the grey zone); these samples were deemed inconclusive. ELISA performance was assessed using a dichotomous approach and compared with respect to sensitivity (Se), specificity (Sp), accuracy, likelihood ratios (LR), and the diagnostic odds ratio (DOR) [32]. The accuracy reflects the capacity of a test in providing correct results and LR, which can be negative or positive, reports how much more likely tested individuals are to have a positive or negative result than non-tested individuals. The higher the positive LR, the more indicative the test is of infection. Conversely, the lower the negative LR, the more significant the test is in ruling out infection. There is a consensus that positive LRs above 10 and negative LRs below 0.1 have a substantial contribution to diagnosis [33]. In the case of a test with 100% specificity, determining the positive LR becomes mathematically impossible since the formula uses the equation “1-specificity” as the denominator. A strategy to overcome this problem is subtracting a value from the sensitivity and specificity values, thus obtaining an estimated result for the positive LR [34]. The DOR was calculated as the ratio between positive and negative LR values. The DOR is considered a global performance parameter that summarizes the diagnostic accuracy of the test. It is a single number that describes the probability of getting a positive result in a person with the disease than in someone without the disease [35]. Confidence intervals (CI) were employed with a confidence level of 95%. Imprecision assessments were based on Cohen’s Kappa coefficient (κ) [36] and the coefficient of variation (CV). The strength of agreement was assessed by κ, with values interpreted as poor (κ ≤ 0), slight (0 <κ ≤ 0.20), fair (0.21 <κ ≤ 0.40), moderate (0.41<κ ≤ 0.60), substantial (0.61 < κ ≤ 0.80) and near perfect agreement (0.81 < κ ≤ 1.0). CV values were calculated as the standard deviation divided by the RI mean. A checklist (S2 Table) and flowchart (S1 Fig) are provided according to the Standards for the Reporting of Diagnostic accuracy studies (STARD) guidelines [37].

## Results

### Diagnostic performance

**Fig 1:**
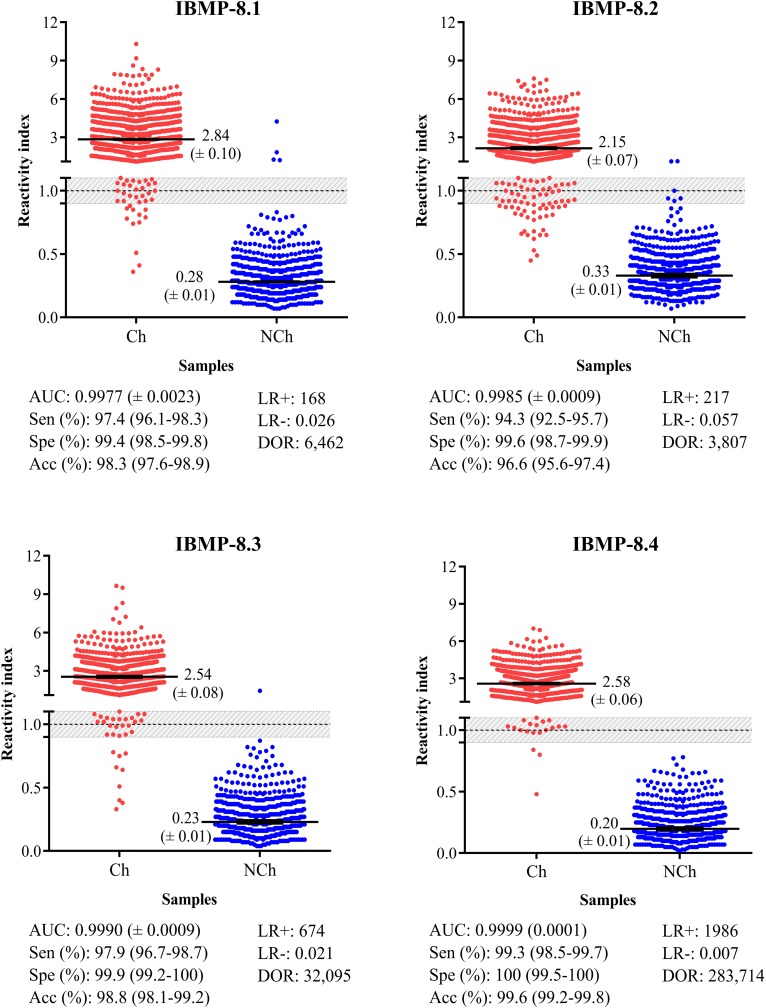
Reactivity index obtained with serum samples from chagasic (Ch) and non-chagasic (NCh) individuals. The cut-off value is reactivity index = 1.0 and the shadowed area represents the grey zone (RI = 1.0 ± 0.10). Horizontal lines and numbers for each group of results represent the geometric means (± 95% CI). AUC (Area Under Curve); Sen (Sensitivity); Spe (Specificity); Acc (Accuracy); LR (Likelihood Ratio); DOR (Diagnostic Odds Ratio).

The assay performance parameters and reactivity index (RI) distributions obtained for the IBMP chimeras are shown in Fig 1 (individual data points are available in the S3 Table). ROC curves were generated from 689 NCh and 857 Ch individuals assayed by ELISA. The AUC (area under the curve) values were 0.9977 for IBMP-8.1, 0.9985 for IBMP-8.2, 0.9990 for IBMP-8.3, and 0.9999 for IBMP-8.4. Except between IBMP-8.2 and -8.4 proteins, the AUC values showed no statistically significant differences. Considering the AUC values, IBMP chimeras exhibited adequate discrimination power and high diagnostic values.

For the panel of Ch sera, IBMP-8.1 produced the highest RI values whereas IBMP-8.2 produced the lowest RI distribution. No significant differences were observed between the RIs of IBMP-8.3 and -8.4. Regarding the NCh samples, the lowest RI intensity was obtained for IBMP-8.4, followed by the IBMP-8.3, -8.1, and -8.2 chimeras.

The IBMP-8.4 chimera produced a sensitivity of 99.3% followed by IBMP-8.3 (97.9%), -8.1 (97.4%), and -8.2 (94.3%). The differences in sensitivity are not statistically significant for the values obtained for IBMP-8.3 and -8.4 or IBMP-8.1 and -8.3. The maximum value of specificity was obtained with the IBMP-8.4 protein (100%). Assays with IBMP-8.3, -8.2, and -8.1 exhibited specificity values of 99.9%, 99.6%, and 99.4%, respectively (Fig 1). The differences between these values are not statistically significant. By adopting RI values of 1.0 ± 0.10 as the grey zone inconclusive interval, we observed that all of the NCh samples fell inside the conclusive space when assayed using the IBMP-8.1, -8.3, and -8.4 chimeric proteins. However, the RI values of four NCh samples (0.58%) fell in the inconclusive zone when tested with the IBMP-8.2 chimera. Regarding the chagasic samples, we observed the following number of samples in the grey zone: 16 (1.87%) assayed with IBMP-8.4, 23 (2.68%) with IBMP-8.3, 24 (2.80%) with IBMP-8.1, and 39 (4.55%) with IBMP-8.2. An overall analysis showed that 1.03% of the samples assayed using IBMP-8.4, 1.49% using IBMP-8.3, 1.55% using IBMP-8.1, and 2.78% using IBMP-8.2 showed RI values falling in the grey zone. The accuracy of the IBMP chimeric proteins was also analyzed. As shown in Fig 1, IBMP-8.4 showed the best accuracy (99.6%) among all the chimeric proteins assayed. IBMP 8–3 also showed high accuracy (98.8%). Lower accuracy values were observed for IBMP-8.1 (98.3%) and IBMP-8.2 (96.6%). The DORs were calculated based on the likelihood ratios. This analysis revealed values of 6,462, 3,807, and 32,095 for IBMP-8.1, -8.2, and -8.3 proteins, respectively. The IBMP-8.4 antigen showed an estimated diagnostic odds ratio of 283,714. Among the chimeric proteins tested, IBMP-8.4 presented the best performance, as noted by the parameters obtained upon ROC analysis, especially regarding the extremely high diagnostic odds ratio shown by this chimera.

### Analysis of antigen sensitivity by geographical origin

In order to assess heterogeneity of recognition of IBMP chimeras by anti-*T*. *cruzi* antibodies due to strain variation and host-related factors, the RI was compared using samples from five Brazilian geographic areas [GO (n = 70), BA (n = 65), MG (n = 58), PR (n = 36), and PE (n = 596)] and from an international commercial panel (n = 32). Differences in sensitivity among all geographical areas are not statistically significant (S2 Fig; individual RI values are given in the S4 Table).

### Analysis of cross-reactivity with other infections

ELISA tests were performed to determine the antigenic cross-reactivity of IBMP proteins against antibodies of unrelated diseases (RI ≥ 1.0) using a panel of 1,079 serum samples. The average RI were very low for all of the antigens tested (IBMP-8.1 = 0.19–0.42, IBMP-8.2 = 0.25–0.41, IBMP-8.3 = 0.17–0.57 and IBMP-8.4 = 0.13–0.28) and the percentage of positive samples was extremely low (S3 Fig; individual RI data points are shown in the S5 Table). Except for the IBMP-8.1 chimera, which showed high RI intensity for samples of two *Leishmania* spp., all other cross-reacting samples produced relatively low RI intensities.

### Assessment of IBMP chimeric proteins combination: Equimolar mixtures

**Fig 2:**
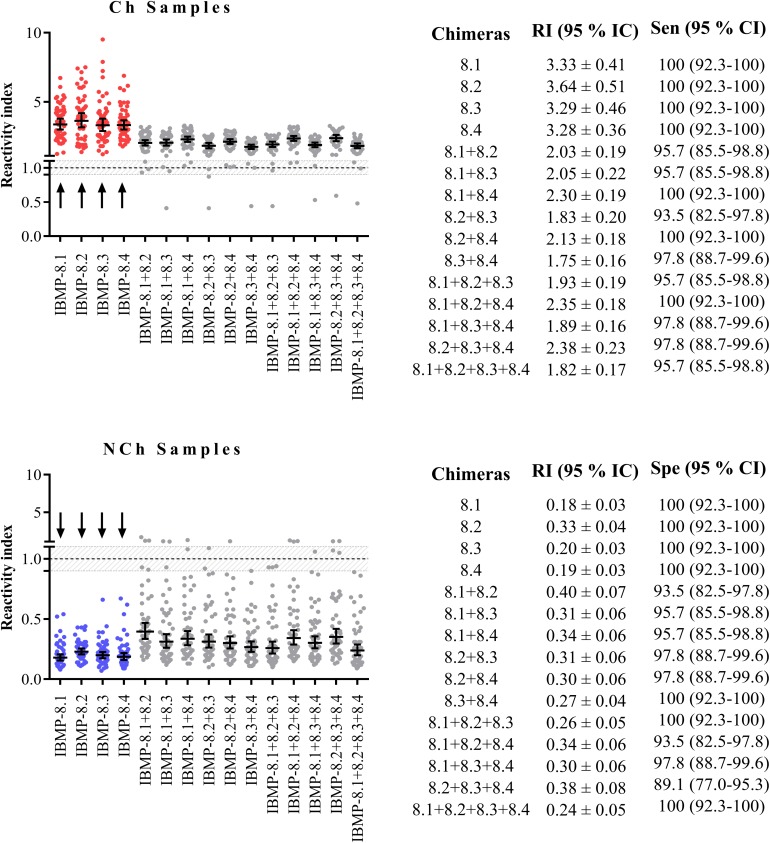
Reactivity index of assays obtained using single (arrows) and equimolar mixtures of IBMP chimeric proteins. The cutoff value was 1.0 and the shadowed area represents the grey zone (RI = 1.0 ± 0.10). Horizontal lines represent the geometric means. RI (Reactivity Index); Sen (Sensitivity); Spe (Specificity); 95% CI (95% Confidence Interval).

The equimolar mixtures of IBMP chimeras were evaluated for serodiagnosis of *T*. *cruzi* to determine whether their combination would improve the diagnostic accuracy. The RI signal of mix-ELISA was compared to the RI signal of tests with single antigens using a panel of 46 sera from chagasic and 46 from non-chagasic patients (individual RI values are available in S6 Table). The sensitivity and specificity of single chimeras were 100% for the chagasic samples while in the equimolar mixtures assays, they ranged from 93.5% to 100% and 89.1% to 100%, respectively (Fig 2). No sample fell into the grey zone when the chimeras were assayed individually. The RI signal obtained by chagasic samples was significantly higher in the assays with individual chimeras relative to the assays using antigen combinations. The opposite was observed for the non-chagasic sera, where the assays with single chimeras (IBMP-8.1, -8.3 or -8.4) resulted in lower RI signal compared to the assays with antigen combinations at equimolar amounts.

### Imprecision assessment

The reliability of diagnostic assays depends on the consistency of its components. In this context, we evaluated assay reproducibility by comparative analyses of experiments within-run, between-lots, and between-labs with a well-defined serum panel from Ch and NCh samples. The evaluated statistical parameters were the coefficient of variation (CV) and Cohen’s Kappa (individual RI values are available in S7 Table). A summary of the coefficient variation analysis for each dataset is shown in Table 1. The coefficient of variation of each antigen was within a 10% range, indicating adequate reproducibility.

**Table 1:** Within-run, between-lots, and between-labs imprecision assessments.

	Within-run			Between-lots			Between-labs		
	Mean RI	SD	CV (%)	Mean RI	SD	CV (%)	Mean RI	SD	CV (%)
**IBMP-8.1**									
Chagasic	2.46	0.139	5.64	3.11	0.245	7.86	2.99	0.228	7.63
Non-Chagasic	0.38	0.030	8.13	0.19	0.018	9.47	0.49	0.022	4.61
**IBMP-8.2**									
Chagasic	2.63	0.232	8.81	3.43	0.225	6.56	1.81	0.117	6.47
Non-Chagasic	0.34	0.028	8.34	0.32	0.019	5.79	0.52	0.049	9.52
**IBMP-8.3**									
Chagasic	3.06	0.175	5.72	3.39	0.321	9.44	2.42	0.112	4.62
Non-Chagasic	0.45	0.034	7.54	0.22	0.021	9.35	0.46	0.043	9.42
**IBMP-8.4**									
Chagasic	2.33	0.214	9.17	3.14	0.257	8.18	2.19	0.179	8.15
Non-Chagasic	0.22	0.019	8.73	0.21	0.019	9.36	0.45	0.024	5.31

Note: RI (Reactivity Index); SD (Standard Deviation); CV (Coefficient of Variation).

**Fig 3:**
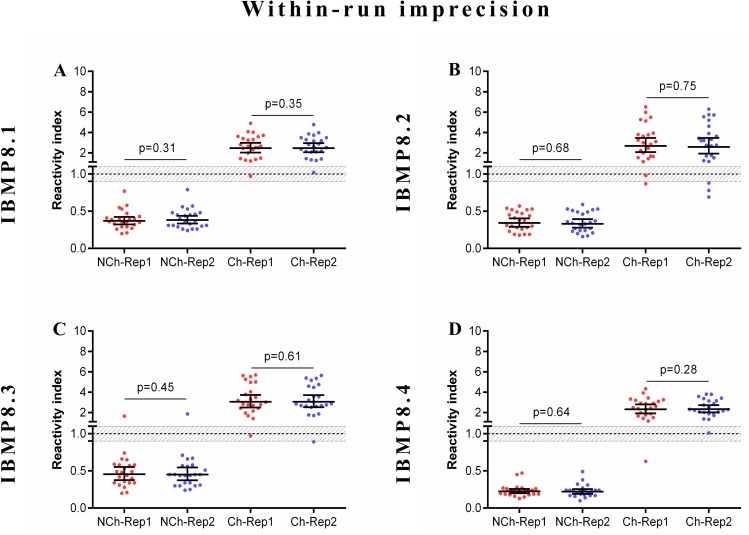
Within-run imprecision analysis. The set of graphs shows reactivity index in chagasic (Ch) and non-chagasic (NCh) serum samples. The cut-off value is 1.0 and shadowed area (RI = 1.0 ± 0.10) represents the grey zone. Horizontal lines and numbers for each group of results represent the geometric means (± 95% CI). Rep (Replicate).

The strength of agreement between the expected results for within-run imprecision varied from 95.7% [κ 0.957 (0.872–1.04)] for IBMP-8.1, -8.2, and -8.4 proteins to 100% for the IBMP-8.3 chimera. As shown in Fig 3, there were no statistically significant differences in RI values between the replicates for Ch and NCh samples.

**Fig 4:**
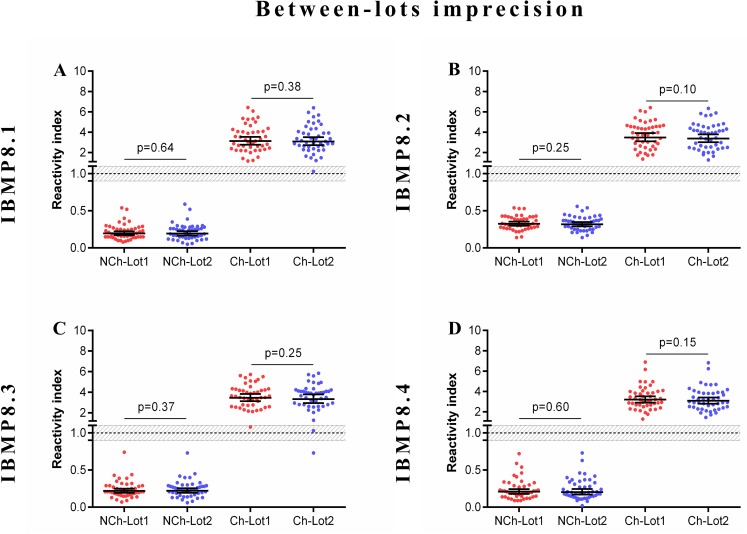
Between-lot imprecision analysis. The set of graphs shows reactivity index in chagasic (Ch) and non-chagasic (NCh) serum samples. The cut-off value was 1.0 and the shadowed area represents the grey zone (RI = 1.0 ± 0.10). Horizontal lines and numbers for each group of results represent the geometric means (± 95% CI).

The overall between-lot agreements were excellent for IBMP-8.1, -8.2 and -8.4, and near perfect for IBMP-8.3 [κ 0.978 (0.935–1.02)]. RI values did not present statistically significant differences between Lot 1 and Lot 2 (Fig 4).

**Fig 5:**
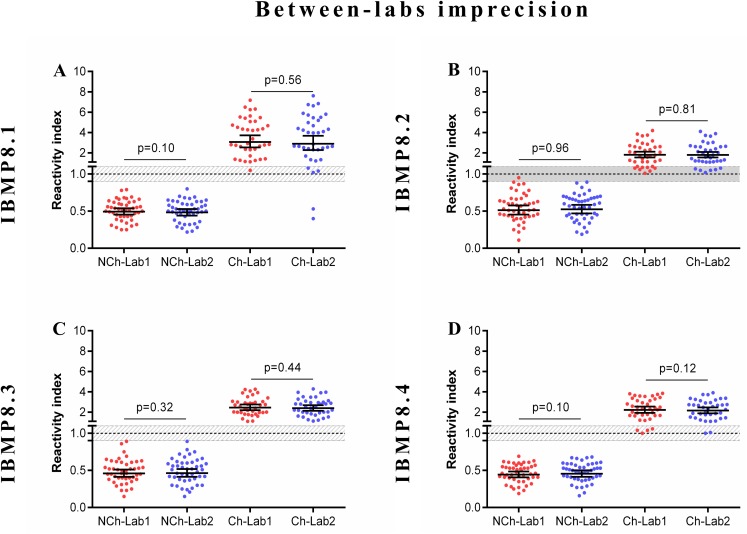
Between-labs imprecision analysis. The set of graphs shows reactivity index in serum samples from chagasic (Ch) and non-chagasic (NCh) individuals. The cut-off value was 1.0 and the shadowed area represents the grey zone (RI = 1.0 ± 0.10). Horizontal lines and numbers for each group of results represent the geometric means (± 95% CI). Lab (Laboratory).

The agreement between Lab 1 and Lab 2 was 95.2% [κ 0.952 (0.886–1.02)] for IBMP-8.1 and 100% for the other chimeras. No statistically significant differences were observed in RI values between the Labs when Ch and NCh samples were assayed (Fig 5).

### Comparison with commercial *T*. *cruzi* ELISA

**Fig 6:**
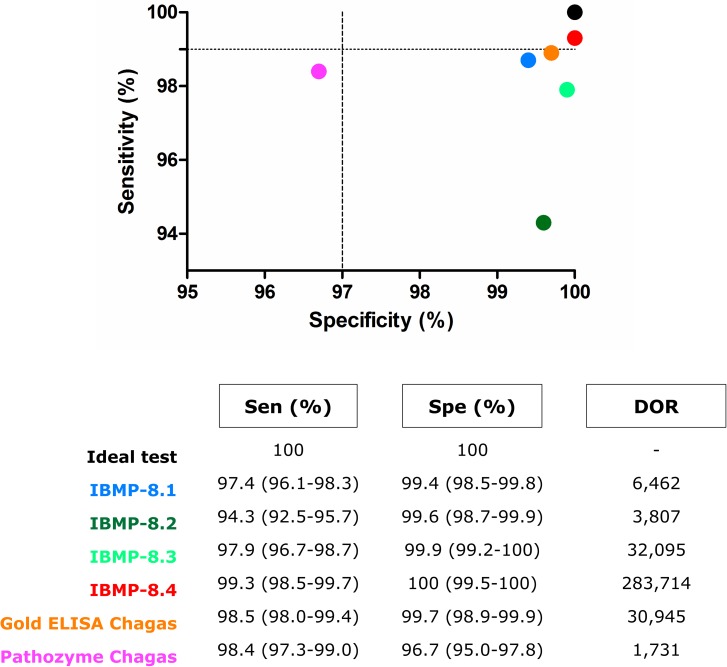
Sensitivity vs. specificity data of IBMP chimeras and two commercial ELISA *T*. *cruzi* assays. The black dot represents an ideal diagnostic test that is 100% accurate.

To compare the accuracy of the IBMP chimeric protein assays with commercial *T*. *cruzi* ELISA we used samples from Ch and NCh individuals. Gold ELISA Chagas and Pathozyme Chagas presented sensitivity of 98.5% (95% CI: 98.0–99.4%) and 98.4% (95% CI: 97.3–99.0%), respectively (Fig 6; individual RI values are available in S8 Table). These values did not differ from that achieved by IBMP-8.1, -8.3, and -8.4 proteins. However, the commercial tests were more sensitive compared to the IBMP-8.2 chimera. Regarding the specificity, the Gold ELISA Chagas and Pathozyme Chagas showed values of 99.7% (95% CI: 98.9–99.9%) and 96.7% (95% CI: 95.0–97.8%), respectively. Specificity of Gold ELISA Chagas did not differ from those found for IBMP chimeras. Regarding the Pathozyme Chagas, the specificity value of this commercial kit was statistically lower than that observed for the IBMP chimeras. The analysis of the DOR values indicates that Gold ELISA Chagas and IBMP-8.3 chimeric protein have similar performance in diagnosing chagasic and non-chagasic patients. However, Pathozyme Chagas presented a lower DOR value compared to IBMP chimeras and the commercial Gold ELISA Chagas.

Fig 6 also shows the relationship between the specificity and sensitivity of IBMP chimeras and commercial ELISA *T*. *cruzi* assays. For comparison purposes, the figure additionally shows the maximum performance of an ideal test (black point 100% accurate). The IBMP-8.4 chimera produced the best results compared to the others chimeras as well as the commercial assays. IBMP-8.4 chimera accuracy is followed by the Gold ELISA Chagas test, and by the IBMP-8.1 and IBMP-8.3 proteins. In contrast, IBMP-8.2 protein presented low sensitivity despite high specificity. Opposite results were observed for the Pathozyme Chagas assay, in which the test produced high sensitivity (>98.0%) and low specificity (<97.0%).

## Discussion

In this study, we assessed the performance of four recombinant proteins for the detection of anti-*T*. *cruzi* antibodies in sera from individuals infected with *T*. *cruzi*, without cross-reaction with sera from control individuals. The assays exhibited a high diagnostic value. In fact, the AUC values were greater than 99%, revealing an optimal discriminative power between negative and chronic CD-positive samples. The antigen IBMP-8.4 is of special interest since it presented an AUC value of 99.99%. These results are in agreement with previous data obtained by our group [9]. Considering the AUC values reported in the literature, the IBMP proteins provided a higher discrimination power compared to other recombinant chimeric proteins, single recombinant proteins, or cell lysates from *T*. *cruzi* strains [38,39]. Furthermore, assays with IBMP proteins yielded a low number of inconclusive results. This was most evident for the IBMP-8.4 chimeric protein. Only 16 out of the 1,546 samples tested with this antigen produced RI values in the grey zone.

The diagnostic sensitivity was higher for IBMP-8.4 compared to the other chimeras and commercial kits. Although there was no statistically significant difference between the IBMP-8.3 protein and commercial kits, the IBMP-8.4 protein assay was the only test with a sensitivity greater than 99%. The IBMP-8.2 protein displayed the lowest sensitivity, probably due its amino acid composition. It is well established that *T*. *cruzi* has high genetic diversity causing discrepant results in serological tests when limited antigens are used. In fact, the parasite is classified into seven genotypes with sub-classifications for regional strains called *clonets* [40–42]. Indeed, 49 Ch samples assayed with IBMP-8.2 were misclassified as negative. From these samples, 44 were derived from distinct Brazilian geographical regions, two from Mexico, two from Nicaragua, and one from Argentina. However, for other proteins, most false-negative results were obtained for samples from Pernambuco, the region with the largest sample contingent. A strategy to overcome this limitation and increase its sensitivity could involve the construction of new molecules using repetitive fragments of other *T*. *cruzi* conserved proteins. Regarding the specificity, all chimeras achieved specificity values above 99%. These data are in agreement with other previous studies [14,28,29]. IBMP specificity values agree with that obtained using the Gold ELISA Chagas and were superior to that achieved using the Pathozyme Chagas assay. This is probably due to the antigens used in this test.

Due to the high proportion of misdiagnosed samples (3.4%) detected using the IBMP-8.2 chimera, its accuracy was significantly lower compared to other proteins. The IBMP-8.4 chimera was found to be 99.6% accurate, suggesting that this antigen can safely be used for chronic CD diagnosis. Although the IBMP-8.1 and -8.3 proteins are less accurate than the IBMP-8.4 chimera, they were found to display values above 98%, indicating that both chimeras can also be used to diagnose chronic CD. The Technical Note N°03/06 from the Brazilian Health Ministry [31] recommends the use of tests with sensitivity and specificity greater than or equal to 99% and 97%, respectively. Based on these recommendations we performed a combined analysis using sensitivity vs. specificity data and observed that only assays using the IBMP-8.4 protein were in accordance with the Technical Note. This finding indicates that the IBMP-8.4 protein may be utilized in serological tests both for *T*. *cruzi* screening in blood banks and for laboratory diagnosis. Blood banks use highly sensitive tests since false negative results may transmit the parasite, and the consequences can be fatal. In contrast, diagnostic tests used for laboratory diagnosis should have high specificity to avoid false-positive results, which could lead to unnecessary treatment, social discrimination, and psychological suffering. Accordingly, if the specificity of IBMP-8.1, -8.2 and -8.3 can be confirmed in further evaluations, they may be useful as antigens for laboratory diagnosis.

Assessment of diagnostic tests using only sensitivity, specificity, and accuracy, as performance parameters, is insufficient to measure their influence on clinical decisions. A diagnostic test will be useful only if its results modify the probability of disease occurrence. Likelihood Ratio (LR) determination is helpful in describing a test’s discriminatory power and defines the probability of a particular result among infected individuals over the probability of this same particular result among healthy individuals [33]. In this study, the IBMP-8.4 showed 100% specificity. Therefore, we subtracted 0.05 from both the sensitivity and specificity values, and obtained an estimated positive LR of 1,986, indicating that a *T*. *cruzi* infected person is approximately 1,986 times more likely to be diagnosed with chronic CD if evaluated with a test using the IBMP-8.4 protein. The estimated DOR for IBMP-8.4 (283,714) was higher than those obtained for IBMP-8.3 (32,095), IBMP-8.1 (6,462), and IBMP-8.2 (3,807). Although the DOR values varied according to the chimera, they were all above 3,500. All the chimeras evaluated in this study performed better than the commercial Pathozyme Chagas. However, the Gold ELISA Chagas showed greater performance than the IBMP-8.1 and -8.2 proteins, but lower than the others. LR and DOR determination are relevant and stable tools in Phase II studies since these parameters do not depend on the prevalence of the disease.

Previous studies have demonstrated that multiantigenic recombinant matrices (mix-ELISA) can enhance the test performance for chronic CD diagnosis compared to the use of individual antigens [11,43,44]. These studies suggest that the advantages of using mix-ELISA are an increase in RI signal, not only for low-reactivity samples, and lack of cross-reactivity with other infections [44]. Another study also demonstrates that the mix-ELISA provides a remarkable decrease in RI signal compared to chimeric constructions [14]. It is proposed that peptides in mixtures adsorbed on solid phase may reduce RI signal by blocking essential chains or competing for binding sites, leading to loss of sensitivity [45–47]. Here, we sensitized microplates using individual chimeras as well as equimolar mixtures compounded by two, three, or four proteins. We assessed the RI signals and performance parameters obtained for negative and chronic CD-positive samples. We preferred to use equimolar mixtures to avoid or minimize blocking of essential chains or competition for binding sites. Our results showed that multiantigenic recombinant matrices increase the RI signal for negative samples and decreases it for chronic CD-positive samples. Additionally, we observed that no sample assayed using individual chimeras fell into the grey zone. Moreover, equimolar mixtures provided false-negative and false-positive results. Based on the above data, we conclude that multiantigenic IBMP matrices of IBMP chimeras impair test performance. Therefore, we show that individual utilization of IBMP chimeras is advantageous due to the cost reduction, simple standardization process, and equilibrated adsorption.

Serological cross-reactivity for IBMP proteins was not surprising as there is a weak seropositivity to unrelated diseases. Indeed, these molecules are composed of specific *T*. *cruzi* fragments, and a modest similarity to human nonpathogenic microorganisms was observed when the amino acid sequences were screened in the NCBI database using the protein BLAST software. It is already well documented that anti-*Leishmania* spp. antibodies are a significant cause of cross-reactivity among serological tests for chronic CD, mainly in conventional tests [9,48–50]. A study in Argentina, for instance, performed in a co-endemic area for leishmaniasis and chronic CD, reported 81.8% (9/11) cross-reactivity using conventional ELISA [51]. A more recent study confirmed this elevated cross-reaction rate and verified a relatively large number of inconclusive results (2.9–8.6%) using conventional and non-conventional tests [9]. Contrary to these data, IBMP-8.1 chimera cross-reacted with two samples and IBMP-8.3 and -8.4 with one sample, demonstrating very little positivity in 153 sample of *Leishmania* spp. Although the *Leishmania* spp. is a concern for accurate chronic CD diagnosis, other unrelated pathogens may also cause cross-reactivity, albeit in minor frequency. Indeed, no cross-reaction was found to dengue, filariasis, HBV, rubella, and measles. For other studied pathogens, the cross-reactivity was insignificant. The number of cross-reacted samples to IBMP chimeras was very small, especially for the IBMP-8.4 protein. Taken together, our data suggest that IBMP chimeras may be used in areas of co-endemicity among *T*. *cruzi* and other diseases.

Part of the process of validating a method to certify that it is appropriate for use is an evaluation of precision, which can be defined as the closeness of agreement between independent measures obtained under identical conditions. While the term precision relates to the concept of variation around a central value, imprecision is really what is measured [52]. Therefore, we used the coefficient of variation (CV) to measure imprecision. For within-run, between-lots, and between-labs analyses we obtained CV values less than 10% for both chagasic and non-chagasic samples, indicating excellent reproducibility both to positive and negative samples. In fact, CV values above 20% typically indicate adequate reproducibility. However, if evidence of excessive variation (>30%) occurs within and between assay runs, more preliminary studies should be performed to verify whether stabilization of the assay is possible or whether the test format should be abandoned [53,54]. Similar results were found by another group that also reported CV lower than 10% [55]. RI values did not vary between measurements, indicating high stability during the reactions. The qualitative assessment of results showed perfect or near perfect agreement using the Cohens’ Kappa method.

The limitations of the study were the exclusion of specimens with discordant results in the commercial tests used to retest the samples that may induce an overestimation of the specificity, and the restricted geographical origin of the tested specimens. Nonetheless, our findings demonstrate that IBMP recombinant chimeras from *T*. *cruzi* presented high overall diagnostic value (accuracy ≥ 95%), and can safely be used as antigens for chronic CD diagnosis. The reported number of cross-reactivity was negligible, thus enabling the use of IBMP chimeras in endemic areas to other infectious diseases, including endemic areas of leishmaniasis. Possible commercial tests using IBMP chimeras will be advantageous due to the cost reduction, simple standardization process, and equilibrated adsorption. IBMP chimeras are quite stable on polystyrene plates, and the results are reproducible. In fact, commercial tests, such as Gold ELISA Chagas, use a mixture of recombinant antigens and lysates from the epimastigotes of *T*. *cruzi* strains, which could lead not only specificity problems but also difficulties in standardizing the method [14]. Based on these results, we will evaluate the performance of IBMP chimeras, notably the IBMP-8.4, using different immunoassay technologies, such as liquid microarray, dual path-platform (DPP), and multiplex ELISA array with positive and negative samples from distinct geographical areas from Brazil and Latin America.

## Supporting information

S1 Table IBMP sequence details. (PDF)

S2 Table STARD checklist. Standards for the Reporting of Diagnostic Accuracy Studies (STARD) checklist for reporting of studies of diagnostic accuracy.
(PDF)

S3 Table Reactivity Index for diagnostic performance assessment. (XLSX)

S4 Table Reactivity Index for performance by geographical origin assessment. (XLSX)

S5 Table Reactivity Index for cross-reactivity assessment. (XLSX)

S6 Table Reactivity Index for equimolar mixtures assessment. (XLSX)

S7 Table Reactivity Index for imprecision assessment. (XLSX)

S8 Table Reactivity Index for comparison with commercial *T. cruzi* ELISA. (XLSX)

S1 Fig STARD flowchart. Standards for the Reporting of Diagnostic Accuracy Studies (STARD) description of the study design.
(TIF)

S2 Fig Reactivity Index and sensitivity for chagasic sera from different geographical areas. The cutoff value is 1.0 and the shadowed area represents the gray zone (RI = 1.0 ± 0.10). Horizontal lines represent the geometric means (± 95% CI). BA (State of Bahia); GO (State of Goiás); ICP (International Commercial Panel); MG (State of Minas Gerais); PE (State of Pernambuco); PR (State of Paraná); RI (Reactivity Index); Sen (Sensitivity); 95% CI (95% Confidence Interval).
(TIF)

S3 Fig Analysis of the cross-reactivity of the IBMP chimeras to sera from unrelated diseases. The cutoff value is 1.0 and the shadowed area represents the gray zone (RI = 1.0 ± 0.10). Horizontal lines represent the geometric means. DENG (Dengue); FILA (Filariasis); HBV (Hepatitis B Virus); HCV (Hepatitis C Virus); HIV (Human Immunodeficiency Virus); HTLV (Human T-cell Lymphotropic Virus); LEIS (Leishmaniasis); LEPT (Leptospirosis); RUBE (Rubella); MEAS (Measles); SCHIS (Schistosomiasis); SYPHI (Syphilis); RI (Reactivity Index).
(TIFF)
