# Supplementary material for: Accuracy of chimeric proteins in the serological diagnosis of chronic chagas disease – a Phase II study
Source: PLoS Negl Trop Dis. 2017 Mar 8;11(3):e0005433. doi: 10.1371/journal.pntd.0005433 (PMC5358787; doi:10.1371/journal.pntd.0005433)
Supplement: S1 Table — (PDF) [file pntd.0005433.s001.pdf]

Constitution of IBMP chimeras

Chimeras	Sequence name	Gene Bank Sequence ID	Amico acid range
IBMP-8.1	Trans-sialidase - *T. cruzi* strain CL Brener	XP_820062.1	747-774
	60S ribosomal protein L19 - *T. cruzi* strain CL Brener	XP_820995.1	218-238
	Trans-sialidase - *T. cruzi* strain CL Brener	XP_813586.1	1435-1449
	Surface antigen 2 (CA-2) - *T. cruzi* strain CL Brener	XP_813516.1	276-297
IBMP-8.2	Antigen, partial - *T. cruzi*	ACM47959.1	13-73
	Surface antigen 2 (CA-2) - *T. cruzi* strain CL Brener	XP_818927.1	166-220
	Calpain cysteine peptidase - *T. cruzi* strain CL Brener	XP_804989.1	31-97
IBMP-8.3	Trans-sialidase - *T. cruzi* strain CL Brener	XP_813237.1	710-754
	Flagellar repetitive antigen protein, partial - *T. cruzi*	AAA30177.1	15-56
	60S ribosomal protein L19 - *T. cruzi* strain CL Brener	XP_808122.1	236-284
	Surface antigen 2 (CA-2) - *T. cruzi* strain CL Brener	XP_813516.1	279-315
IBMP-8.4	Shed-acute-phase-antigen - *T. cruzi*	CAA40511.1	681-704
	Kinetoplastid membrane protein KMP-11 - *T. cruzi* strain CL Brener	XP_810488.1	76-92
	Trans-sialidase - *T. cruzi* strain CL Brener	XP_813586.1	1436-1449
	Flagellar repetitive antigen protein - *T. cruzi*	AAA30177.1	20-47
	Trans-sialidase - *T. cruzi* strain CL Brener	XP_820062.1	740-759
	Surface antigen 2 (CA-2) - *T. cruzi* strain CL Brener	XP_813516.1	276-298
	Flagellar repetitive antigen protein, partial - *T. cruzi*	AAA30197.1	1-68
	60S ribosomal protein L19 - *T. cruzi* strain CL Brener	XP_820995.1	218-238
	Microtubule-associated protein - *T. cruzi* strain CL Brener	XP_809567.1	421-458
