# Supplementary figures and images for: Accuracy of chimeric proteins in the serological diagnosis of chronic chagas disease – a Phase II study
Source: PLoS Negl Trop Dis. 2017 Mar 8;11(3):e0005433. doi: 10.1371/journal.pntd.0005433 (PMC5358787; doi:10.1371/journal.pntd.0005433)

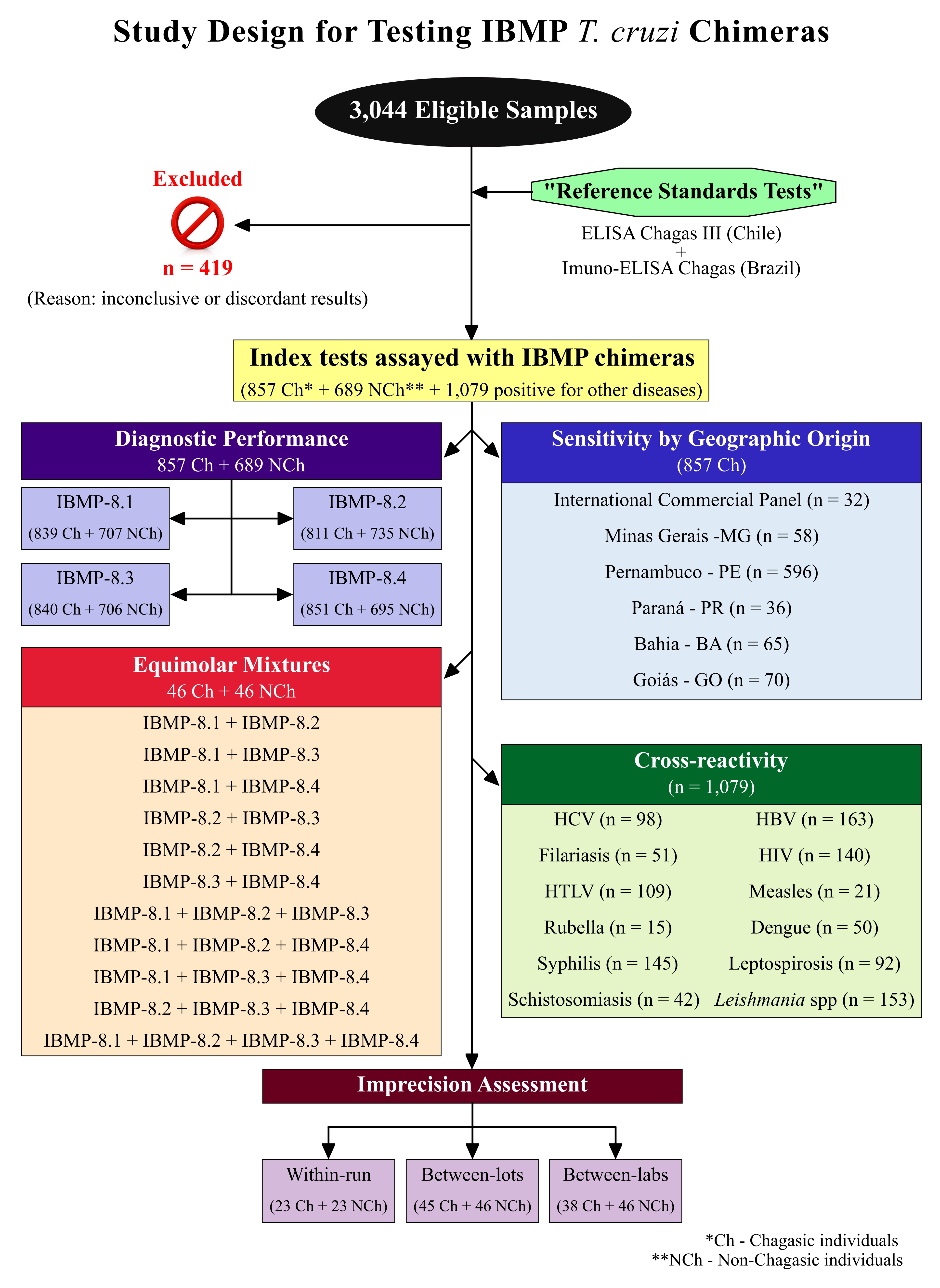

Supplement: S1 Fig — Standards for the Reporting of Diagnostic Accuracy Studies (STARD) description of the study design. (TIF) [file pntd.0005433.s009.tif]

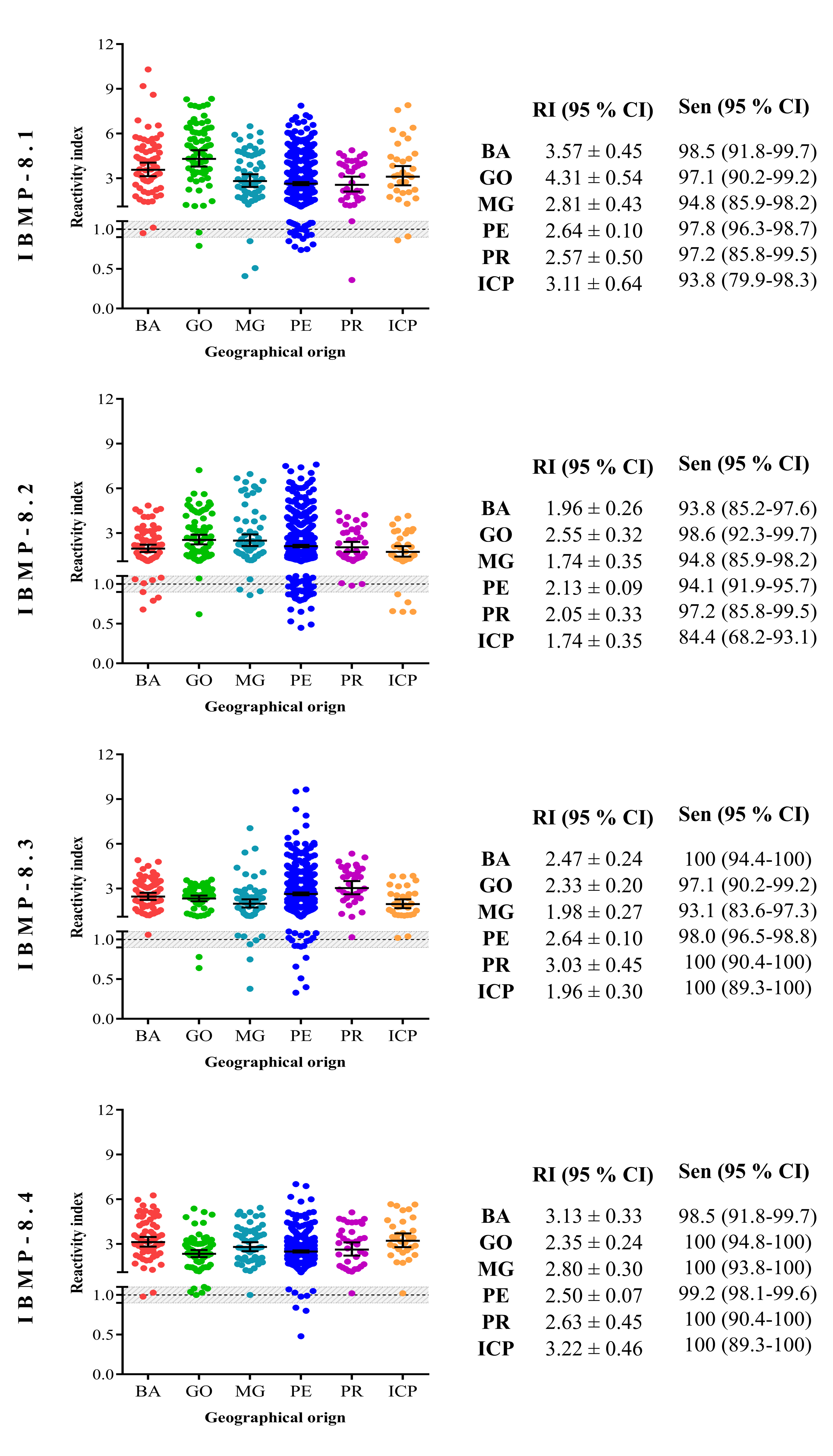

Supplement: S2 Fig — The cutoff value is 1.0 and the shadowed area represents the gray zone (RI = 1.0 ± 0.10). Horizontal lines represent the geometric means (± 95% CI). BA (State of Bahia); GO (State of Goiás); ICP (International Commercial Panel); MG (State of Minas Gerais); PE (State of Pernambuco); PR (State of Paraná); RI (Reactivity Index); Sen (Sensitivity); 95% CI (95% Confidence Interval). (TIF) [file pntd.0005433.s010.tif]

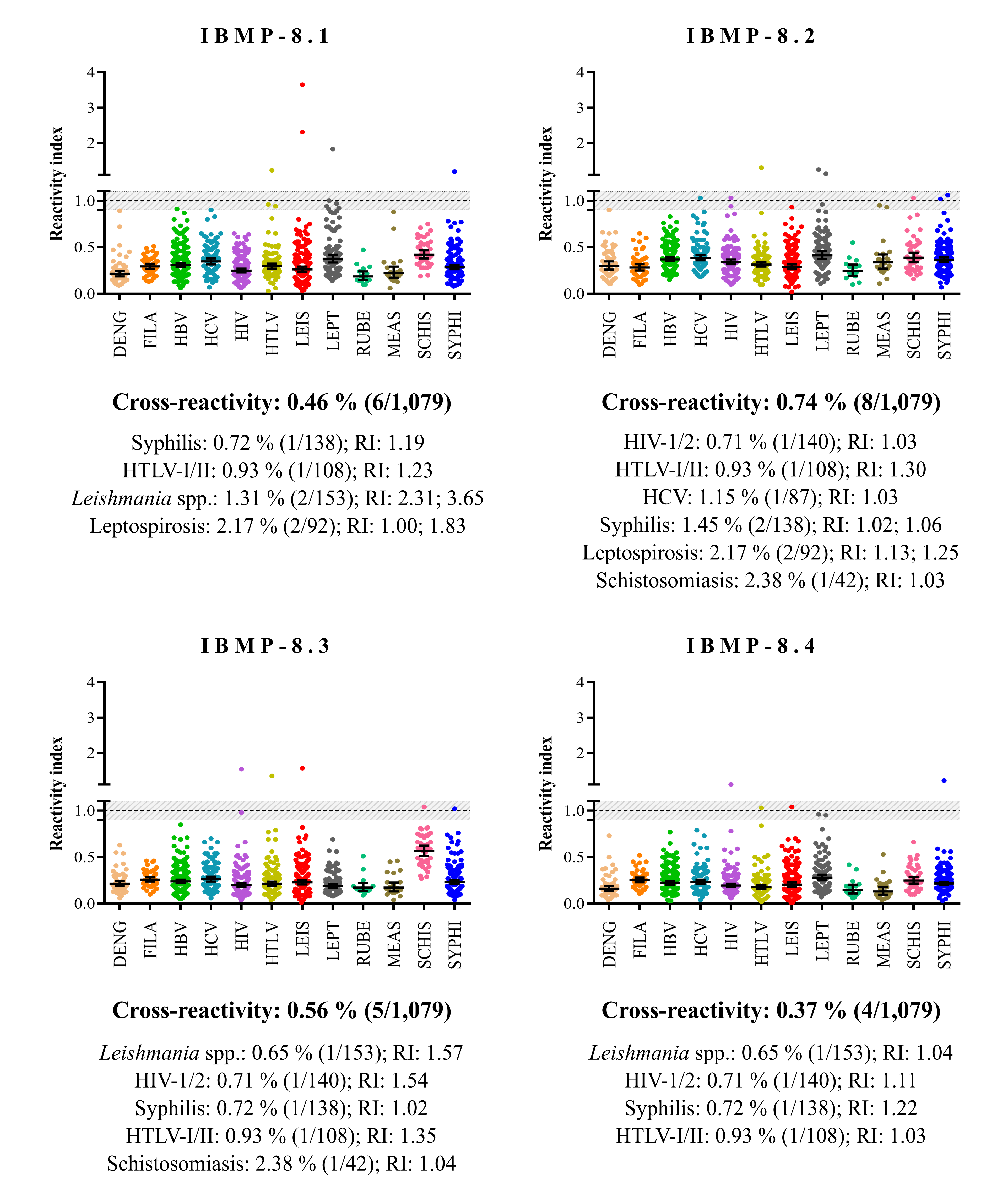

Supplement: S3 Fig — The cutoff value is 1.0 and the shadowed area represents the gray zone (RI = 1.0 ± 0.10). Horizontal lines represent the geometric means. DENG (Dengue); FILA (Filariasis); HBV (Hepatitis B Virus); HCV (Hepatitis C Virus); HIV (Human Immunodeficiency Virus); HTLV (Human T-cell Lymphotropic Virus); LEIS (Leishmaniasis); LEPT (Leptospirosis); RUBE (Rubella); MEAS (Measles); SCHIS (Schistosomiasis); SYPHI (Syphilis); RI (Reactivity Index). (TIFF) [file pntd.0005433.s011.tiff]
